# *GmABR1* encoding an ERF transcription factor enhances the tolerance to aluminum stress in *Arabidopsis thaliana*

**DOI:** 10.3389/fpls.2023.1125245

**Published:** 2023-03-23

**Authors:** Hongjie Wang, Cheng Li, Lidan Wang, Hongying Zhong, Xin Xu, Yanbo Cheng, Hai Nian, Wenhua Liu, Pei Chen, Aixia Zhang, Qibin Ma

**Affiliations:** ^1^The State Key Laboratory for Conservation and Utilization of Subtropical Agro-bioresources, South China Agricultural University, Guangzhou, China; ^2^The Guangdong Province Key Laboratory of Plant Molecular Breeding, College of Agriculture, South China Agricultural University, Guangzhou, China; ^3^The Guangdong Subcenter of the National Center for Soybean Improvement, College of Agriculture, South China Agricultural University, Guangzhou, China; ^4^The Guangdong Provincial Laboratory of Lingnan Modern Agricultural Science and Technology, South China Agricultural University, Guangzhou, China; ^5^Zengcheng Teaching and Research Bases, South China Agricultural University, Guangzhou, Guangdong, China; ^6^Agro-biological Gene Research Center, Guangdong Academy of Agricultural Sciences, Guangzhou, Guangdong, China

**Keywords:** *GmABR1*, soybean, Al stress, transgenic *Arabidopsis thaliana*, transcription factor

## Abstract

The ethylene response factor (ERF) transcription factors, which is one of the largest transcription factor families in plants, are involved in biological and abiotic stress response and play an important role in plant growth and development. In this study, the *GmABR1* gene from the soybean inbred line Zhonghuang24 (ZH24)×Huaxia 3 (HX3) was investigated its aluminum (Al) tolerance. GmABR1 protein has a conserved domain AP2, which is located in the nucleus and has transcriptional activation ability. The results of real-time quantitative PCR (qRT-PCR) showed that the *GmABR1* gene presented a constitutive expression pattern rich in the root tip, stem and leaf tissues of HX3. After Al stress, the *GmABR1* transcript was significantly increased in the roots. The transcripts of *GmABR1* in the roots of HX3 treated with 50 µM AlCl_3_ was 51 times than that of the control. The *GmABR1* was spatiotemporally specific with the highest expression levels when Al concentration was 50 µM, which was about 36 times than that of the control. The results of hematoxylin staining showed that the root tips of *GmABR1*-overexpression lines were stained the lightest, followed by the control, and the root tips of *GmABR1* RNAi lines were stained the darkest. The concentrations of Al^3+^ in root tips were 207.40 µg/g, 147.74 µg/g and 330.65 µg/g in wild type (WT), overexpressed lines and RNAi lines, respectively. When AlCl_3_ (pH4.5) concentration was 100 µM, all the roots of Arabidopsis were significantly inhibited. The taproot elongation of WT, *GmABR1* transgenic lines was 69.6%, 85.6%, respectively. When treated with Al, the content of malondialdehyde (MDA) in leaves of WT increased to 3.03 µg/g, while that of transgenic Arabidopsis increased from 1.66-2.21 µg/g, which was lower than that of WT. Under the Al stress, the Al stress responsive genes such as *AtALMT1* and *AtMATE*, and the genes related to ABA pathway such as *AtABI1*, *AtRD22* and *AtRD29A* were up-regulated. The results indicated that *GmABR1* may jointly regulate plant resistance to Al stress through genes related to Al stress response and ABA response pathways.

## 1 Introduction

Aluminum (Al) third only to oxygen and silicon is one of the most abundant metallic elements in the earth’s crust. When the soil pH is below 5.5, Al is mainly present in the form of Al^3+^ which is highly toxic to plants (Ranjan et al., 2021). Approximately 50% of the world’s arable soils are acidic, and Al toxicity is a major factor limiting crop yields under abiotic stress in acidic soils (Kochian et al., 2015). The Al absorbed by plants is first concentrated in the root tips. Previous studies have found that the Al^3+^ content of root tip meristem in Al-tolerant plants is generally lower than that of Al-sensitive plants (Rangel et al., 2009). After some heavy metal ions enter root cells of plants, enzyme activity is inhibited and plant metabolism is blocked (Chen, 2006). The elongation and division of root tip cells were severely inhibited under the action of Al^3+^. The root tip enlargement, main root coarsening and shortening, lateral root number decreased correspondingly. The photosynthesis of leaves was inhibited with yellowing and curling shape, and smaller leaf area (Chauhan et al., 2021). The root changes of plants may be due to the inhibition of cellulose synthesis in root tip cell wall by Al^3+^ (Matsumoto and Motoda, 2012). Plants exposed to Al for a long time will be inhibited the absorption of water and other nutrients in their roots resulting in plant production and even death (Zhang et al., 2019a).

AP2/ERF transcription factors play an important role in plant abiotic stress. They can specifically bind *cis*-elements in the promoter region of downstream defense genes including activating or inhibiting the expression of downstream stress-responsive genes through positive and negative regulation, and thus participate in the response to plant stress (Kumar and Sharma, 2021). The AP2/ERF transcription factor contains one or two conserved AP2/ERF domains consisting of approximately 58-70 amino acids which are involved in DNA binding (Li et al., 2022). The AP2/ERF domain consists of three β folds and one amphiphilic α helix. There are differences between the two amino acid residues at position 14 and 19 in the second β fold, which makes the AP2/ERF transcription factor specific to bind different *cis*-acting elements (Feng et al., 2020).

The AP2/EREBP transcription factor OPBP1 can regulate the expression of stress-related genes. The overexpression of *OPBP1* in tobacco plants showed strong resistance to *Pseudomonas syringae*pv *tabaci* and *Phytophthora parasiticavar nicotianae* pathogens, and improved the resistance to salt stress (Guo et al., 2004). Soybean gene *GmERF5* was involved in the response to *Phytophthora sojae* infection. The tolerance of transgenic lines to *Phytophthora sojae* infection was significantly improved compared with the control. *GmERF5* was significantly induced. While its promoter activity was activated under the treatments of ethylene (ET), abscisic acid (ABA), *Phytophthora sojae*, salt and drought. The results suggested that *GmERF5* was not only involved in the induced defense response but also involved in ABA-mediated pathways of salt and drought tolerance (Dong et al., 2015). In rice, *OsERF922* was rapidly and strongly up-regulated by ABA, salt and blast fungus. *OsERF922* knockout by RNAi enhanced rice resistance to *magnaporthe oryzae* and increased the expression of several genes regulating plant stress resistance synthase. In contrast, plants that overexpressed *OsERF922* reduced the expression of these defense-related genes and increased susceptibility to *magnaporthe oryzae*. The ABA content of *OsERF922* overexpressed rice was significantly higher than that of control and RNAi lines. *NCED3* and *NCED4* were up-regulated in *OsERF922* overexpressed plants, while *NCED4* was down-regulated in RNAi lines. *OsERF922* may enhance plant resistance to abiotic stress by participating in the ABA pathway (Liu et al., 2012). Abscisic acid repressor ABR1, a member of ethylene responsive element binding factor superfamily of AP2/ERF transcription factors, significantly improves drought resistance of rice, crop quality and yield (Pandey et al., 2005). *Fagopyrum esculentum* can cope with aluminum toxicity by increasing ABA level and antioxidant system in early growth and development. The levels of reactive oxygen species (ROS), endogenous ABA and antioxidant enzyme activity in root of buckwheat seedlings increased after aluminum treatment, which may be mediated by increased ABA levels. After ABA treatment, ROS levels increased, catalase (CAT) and ascorbate peroxidase (APX) activities increased (Salazar-Chavarria et al., 2020). *OsERF71* plays a positive role in drought tolerance of rice by increasing the expression of genes related to ABA signaling pathway and proline synthesis. Overexpression of *OsERF71* decreased the water loss rate of rice, while the RNAi lines of *OsERF71* were sensitive to drought stress and increased the water loss rate. Under drought stress, *OsERF71* regulated the expression of several ABA responsive genes and proline synthesis genes, enhanced the sensitivity and proline accumulation of ABA treatment. *GsERF71* overexpressing Arabidopsis has significantly higher tolerance to carbonate stress induced by NaHCO_3_ or KHCO_3_ than that of wild-type plants with greener leaves, longer roots, higher chlorophyll content and lower MDA content. *GsERF71* increased the tolerance to alkaline stress by up-regulating H^+^, ATPase expression and regulating auxin accumulation in transgenic plants (Yu et al., 2017). SHINE (SHN) transcription factor is a branch of AP2/ERF and one of the first reported transcription factors to regulate plant cuticle formation. Cuticle plays an important role in improving plant abiotic stress, such as drought resistance, high temperature resistance, salt resistance, disease resistance (Khoudi, 2023). Recent study showed that the *GsERF1* gene from BW69 wild soybean was induced by aluminum stress and ethylene (Li et al., 2022).

The quality and yield of soybean are greatly affected and limited by acidic soil in a large area of the world. In the past, there were a few reports on the involvement of ERF family genes in Al tolerance in plants. In this study, the ethylene reactive transcription factor *GmABR1* of AP2/ERF family gene was identified using the population of recombinant inbred line derived from Zhonghuang 24 (ZH24) × Huaxia 3 (HX3), and its function tolerant to Al stress was investigated by heterotransformation of Arabidopsis.

## 2 Materials and methods

### 2.1 Plant materials and growth conditions

In this experiment, the HX3 soybean cultivar was bred and provided by the Guangdong Subcenter of National Soybean Improvement Center. The tissue expression pattern of *GmABR1* and its response to Al stress were studied using qRT-PCR. The seeds of HX3 were germinated on vermiculite after high temperature sterilization. The culture temperature was 26 °C and the light/darkness was 16 h/8 h (Wang et al., 2019a). The cultures were incubated in 0.5 mM CaCl_2_ solution (containing 50 µM AlCl_3_) for 0, 3, 6, 9, 12, 24, 36, 48, 60 and 72 h. With the incubation in 0.5 mM CaCl_2_ with 0, 25, 50, 75, or 100 µM AlCl_3_ (pH4.5) for 24 h, all the roots of soybean are taken as samples in Al dose-effect experiments (Han et al., 2022). For tissue expression pattern, the samples of roots, stems, and leaves of soybean seedlings incubated in 0.5 mM CaCl_2_ (50 µM AlCl_3_ (pH4.5) for the experimental group, without AlCl_3_ for the control group) for 60 h were taken and rapidly frozen in liquid nitrogen, and stored at -80°C (Wang et al., 2022).

### 2.2 Bioinformatics analysis and cloning of the *GmABR1* gene

The *GmABR1* gene in soybean cultivar HX3 was up-regulated in the expression profile of acid-resistant aluminum genes, and the login number on the website of the National Center for Biotechnology Information (NCBI) was XP_003523249.1 by using the NCBI database for searching for the sequence information of the candidate gene. The software DNAMAN 9.0 was used for sequence alignment, phylogenetic tree was constructed by proximity method (NJ) using MAGE 7.0 software (Shu et al., 2022). The total RNA of soybean cultivar HX3 extracted by *Tri*zol solution (Vazyme Biotech Co., Ltd., Nanjing, China) was reverse-transcribed into cDNA (HiScript^®^ III RT SuperMix for qPCR, Vazyme Biotech Co., Ltd.; Nanjing, China) (Wang et al., 2022). The full-length of *GmABR1* gene was cloned by using specific primers and high-fidelity DNA polymerase (Phanta Max, Vazyme Biotech Co., Ltd.; Nanjing, China) (**Table S2**). PCR products were detected by 1% agarose gel electrophoresis (GenStar Kit, GenStar Development Company, Calgary, AB, Canada), and the purified PCR products were inserted into the polyclonal site of the zero-background clone pLB vector (Tiangen Lethal Based Fast Cloning Kit, Beijing, China). The fusion vector GmABR1-pLB was transformed into *Escherichia coli* DH5α (Li et al., 2022). The positive clones of *GmABR1* gene were identified by PCR and enzyme digestion, and the CDS sequence of *GmABR1* gene was obtained by sequencing [Sangon Biotech (Shanghai) Co., Ltd., China] (Wen et al., 2021).

### 2.3 Construction of *GmABR1* overexpression vector and genetic transformation of Arabidopsis

Using the constructed pLB plasmid as DNA template, the CDS sequence of *GmABR1* amplified by PCR was inserted between the restriction sites of *Xba*I and *Sac*I from the vector pTF101 by using the ClonExpressR II One Step Cloning Kit (C112, Vazyme, Nanjing, China) to construct the vector pTF101-GmABR1. The positive clones of pTF101-GmABR1 were identified by PCR using specific primers. The pTF101-GmABR1 plasmid was transformed into *agrobacterium tumefaciens* GV3101 cells by heat shock method (Li et al., 2022). Genetic transformation of Arabidopsis was subsequently performed using agrobacterium-mediated flower impregnation (Han et al., 2022). After the obtained transgenic plants were identified, the T_3_ transgenic positive lines with high expression of *GmABR1* in root tip were selected for subsequent experiments including the analysis of its expression patterns response to Al stress and the phenotypes resistant to Al stress (Liu et al., 2020). The primer information used in this experiment was shown in **Table S2**.

### 2.4 Subcellular localization of GmABR1 protein

The subcellular localization of GmABR1 protein was analyzed using the method described by Collings et al. and Dong et al. (Collings, 2013; Dong et al., 2015). The subcellular localization of GmABR1 protein was predicted using a web server Cell-PLoc 2.0 (http://www.csbio.sjtu.edu.cn/bioinf/Cell-PLoc-2/). The specific operation is to cut the *Nco*I and *Spe*I sites on the expression vector pCAMBIA1302-eGFP with restriction endonuclease, and to link the recombinant amplified fragments of CDS sequences containing corresponding restriction sites with T4 ligase. After the construction of the fusion expression vector, the plasmid pCAMBIA1302-eGFP-GmABR1 and the empty plasmid pCAMBIA1302-eGFP were transformed into the cells of *Agrobacterium tumefaciens* GV3101 (contain the virus protein P19) and then injected into the lower epidermal cells of 4-week-old leaf in *Nicotiana tabacum*. The transformed leaves for 48 hours were observed with a laser confocal microscope (Carl Zeiss, Jena, Germany).

### 2.5 Transactivation assay of GmABR1 protein

The activation activity of GmABR1 protein was verified by using the method described by Han et al. (Han et al., 2022). The full-length sequence of *GmABR1* was first cloned and inserted it into the *Bam*HI and *Eco*RI restriction endonuclease sites of pGBKT7 vector to form the fusion expression vector pGBKT7-GmABR1. The vectors of pGBKT7 and pGBKT7-GmABR1 were then transformed into yeast Y_2_H strain, respectively. The transformed yeast cells were cultured on SD medium which was lacking Trp (SD/-Trp) at 30 °C for 3 days. Then the positive colonies were transferred from SD/-Trp medium to SD/-Trp medium containing 125 μg/L X-α-gal. After cultured for 3 days, whether the colonies appear blue or not can be observed on the selected medium.

### 2.6 Gene expression analysis by qRT-PCR

Total RNA of samples was extracted from soybean or Arabidopsis tissues using the *Trizol* solution (Vazyme Biotech Co., Ltd., Nanjing, China). qRT-PCR and first strand cDNA synthesis templates were performed with 1 μg of RNA using the HiScriptII first strand cDNA synthesis kit (Vazyme Biotech Co., Ltd., Nanjing, China) (Cai et al., 2018). qRT-PCR was performed using SYBR PremixEx-Taq™ II (Takara Bio Inc., Kusatsu, Japan) and real-time CFX96TM systems (Bio-Rad, Hercules, CA, USA) to detect RNA transcripts of related genes (Wang et al., 2022). The inner reference gene *Actin3* in soybean was used to normalize the data. The expression data were calculated using the comparative cycle threshold method 2^-△△ct^. The experiments were repeated at three independent organisms (Xian et al., 2020). The primers used in this assay were listed in **Table S2**.

### 2.7 Phenotypic analysis of root growth in Arabidopsis treated with Al

Three homozygous lines from T_3_ generation of *GmABR1* transgenic plants were used to measure taproot length as described previously (Yokosho et al., 2016; Li et al., 2022). The seeds of *GmABR1* transgenic Arabidopsis and WT were disinfected with 10% sodium hypochlorite for 10 min respectively, during which they were vortically oscillated, and then cleaned with sterilized water for 5 times. The sterilized seeds were sown on a square plate of 1/2 MS solid medium (pH5.8), placed in a refrigerator at 4°C and vernalized at low temperature for 3-4 d. Then the seeds were germinated and grown in the growth chamber with following conditions: 23°C, 60% relative humidity, 100 mol photons m^-2^·s^-1^, 16 h light and 8 h dark cycles. When root lengths of the seedlings grew to 1-2 cm, the seedlings were selected and transferred to the medium containing different concentrations of AlCl_3_ (0, 25, 50 and 100 μM; pH 4.5). Phenotypic differences were observed and photographed after 3 days of Al treatment. The data were analyzed by using the software Image J (Zhou et al., 2008).

### 2.8 Soybean hairy root transformation and hematoxylin staining

The construction of pMU103-GmABR1 interference vector (RNAi vector) is similar to the previous construction method (Kereszt et al., 2007; Ren et al., 2019). The 159 bp coding region sequence of *GmABR1* gene was selected to construct the interference vector. The vector plasmids of pTF101, pTF101-GmABR1, pMU103-GmABR1 were transformed into *Agrobacterium rhizogenes* K599 competent cells by heat shock, respectively. Soybean hair roots from the 5-day’s seedlings were used as experimental materials, and then transformed by the method of hypocotyl injection.

In order to analyze the expression of *GmABR1* gene in soybean hairy roots, the hairy root lines were treated with 50 µM AlCl_3_ solution (CaCl_2_, pH 4.5) for 6 h, then the roots were cut and dipped into hematoxylin solution for 30 min. The stained roots were taken out and rinsed for 30 min before being observed under stereomicroscope (Li et al., 2022).

### 2.9 Physiological indices assay

Transgenic *GmABR1* Arabidopsis and WT were treated with 0.5 mM CaCl_2_ solution containing 50 µM AlCl_3_ and 0.5 mM CaCl_2_ solution without AlCl_3_ (pH 4.5), respectively. After 10 days, the whole plant samples were taken to determine the content of MDA (Choudhury and Sharma, 2014).

The hairy root lines transferred into pTF101 plasmid were used as control. The roots of WT, *GmABR1-*overexpressed soybean and *GmABR1-*RNAi soybean were transferred to 0.5 mM CaCl_2_ solution (pH 4.5) containing 50 µM AlCl_3_. After 1 day of treatment, the root tips of 2 cm washed with ddH_2_O were taken as samples, weighed and recorded. Put 2 M hydrochloric acid (HCl) into a 2 ml EP tube and soak it in a shaking table for 3-4 days. After extraction and filtration, the content of Al^3+^ in soybean hairy roots was determined by plasma inductances coupled (Umemura et al., 2003).

### 2.10 Statistical analyses

Each experiment was designed at least three biological replicates. The SPSS Statistics (v.22) and GraphPad Prism 8 softwares were used to analyze and process the data. The statistical significance of the difference for the data was indicated by an asterisk (*P < 0.05; **P < 0.01) (Ma et al., 2018; Wang et al., 2022).

## 3 Results

### 3.1 Isolation and bioinformatics analysis of *GmABR1*

**Figure 1:**
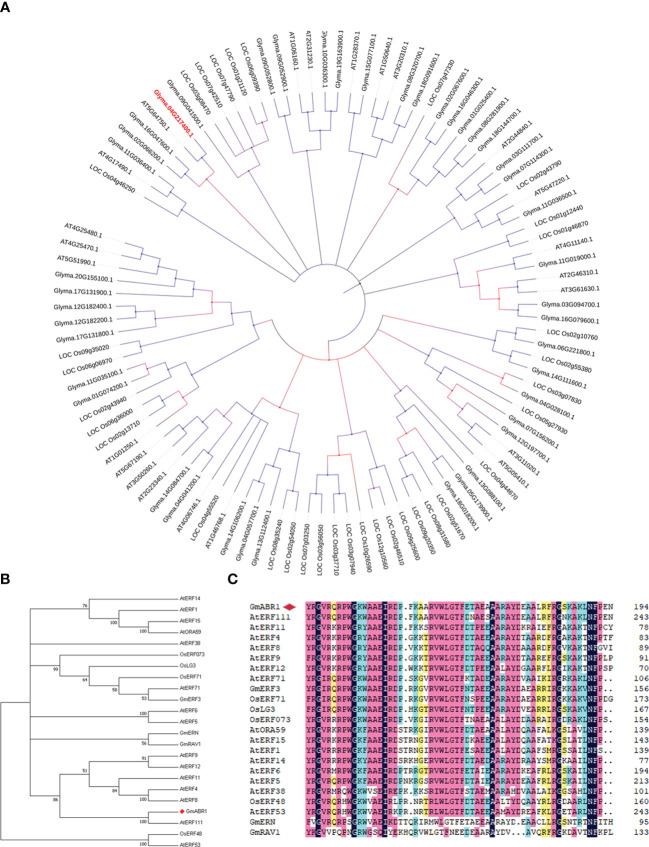
Amino acid sequence and phylogenetic analysis of ERF transcription factors. **(A)** Phylogenetic tree analysis of GmABR1 and ERF proteins in representative species of major lineage of plants. **(B)** Phylogenetic analysis of ERF proteins in higher plants. **(C)** Sequence alignment of the AP2 domain in ERF proteins of higher plants. The comparison of amino acid sequences was conducted by the software of DNAMAN9.0. The phylogenetic tree was constructed by the neighbor-joining method using the software of MEGA 7.0. The above involves the amino acid sequences of ERF family from the NCBI database (https://www.ncbi.nlm.nih.gov/). The detailed information of ERF proteins was available from the **Table S3**.

The *GmABR1* gene was cloned from soybean cultivar HX3 using specific primers (**Table S1**). The *GmABR1* gene sequence was obtained from the National Center for Biotechnology Information (NCBI) database with the accession number LOC_100793215. The length of *GmABR1* gene was 1135 bp, and the length of open reading frame was 846 bp. It contains two exons encoding a 282 amino acid protein with an estimated molecular weight of 30.8504 KDa and isoelectric point of 10.0135. Sequence alignment revealed that GmABR1 protein had a conserved DNA-binding domain (AP2 domain) consisting of 60 amino acids between 135-194 amino acid residues, which was inferred to be a typical structure of ERF transcription factors. In plants, many ERF family genes have been reported to be closely associated with biotic and abiotic stresses. Amino acid sequence alignment and phylogenetic tree analysis showed that GmABR1 protein had the highest homology up to 93% with AtERF111 (*At5g64750*) protein in Arabidopsis (**Figure 1**).

### 3.2 Subcellular localization and transcriptional activation ability of GmABR1 protein

**Figure 2:**
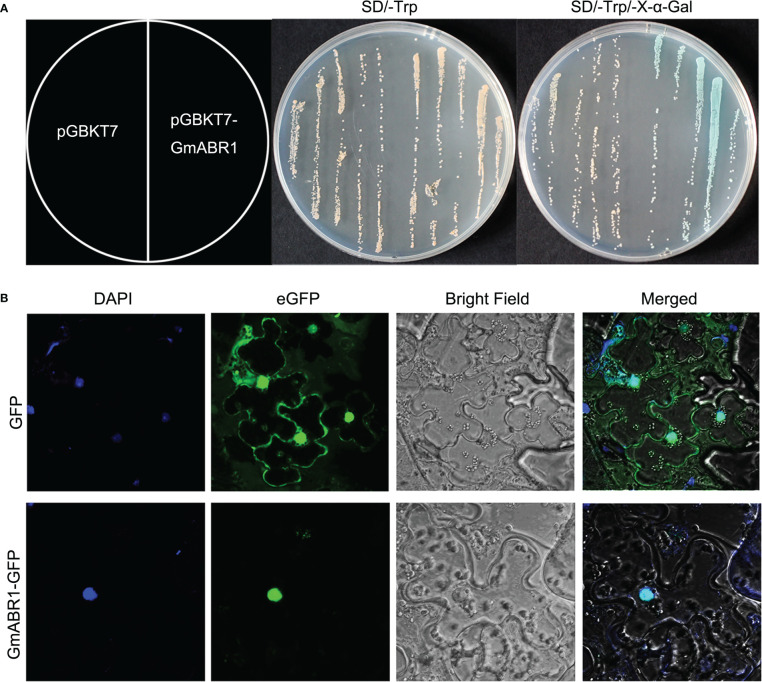
Transcriptional activity detection and subcellular localization of GmABR1 protein. **(A)** Transcriptional activation of GmABR1 protein. **(B)** Subcellular localization of GmABR1 protein. The CDS of *GmABR1* sequence was fused with the GAL4 DNA binding region of pGBKT7 vector and expressed in yeast strain Y_2_H Gold. After the transformation, the two positive clones were transferred to SD/-TRP solid medium, and the other group was added with substrate X-a-Gal. The pCAMBIA1302-GFP-GmABR1 and pCAMBIA1302-GFP vectors were transformed into *Agrobacterium tumefaciens* GV3101 (contain the virus protein P19) respectively, and then injected into 4-week old *Nicotiana tabacum* L epidermal cells. After cultured for 2 days later, the leaves were observed by laser confocal microscopy (Carl Zeiss, Jena, Germany).

We detected the localization of GmABR1 protein in tobacco leaf cells. The results of confocal laser microscopy showed that GFP protein stimulated green fluorescence in the whole cell, while GmABR1-GFP fusion protein only stimulated green fluorescence in the nucleus, which overlapped with the chromophore of nuclear dye DAPI (**Figure 2B**). The results showed that the GmABR1 protein is localized in the nucleus.

To determine the transcriptional activity of GmABR1 protein, we combined the ORF of GmABR1 with the pGBKT7 plasmid, and simultaneously transferred the constructed fusion plasmid pGBKT7-GmABR1 and pGBKT7 vector into the receptive cells of yeast strain Y_2_H. The results showed that the transformed yeast positive clones of pGBKT7-GmABR1 and pGBKT7 plasmids could grow on SD/-Trp medium. In addition, the yeast monoclonal transformed with pGBKT7-GmABR1 plasmid was blue on SD/-Trp-X-α-Gal solid medium, while the yeast monoclonal transformed with pGBKT7 vector showed no color change (**Figure 2A**). The results showed that GmABR1 protein had characteristic transcriptional activation.

### 3.3 Expression patterns of *GmABR1*

**Figure 3:**
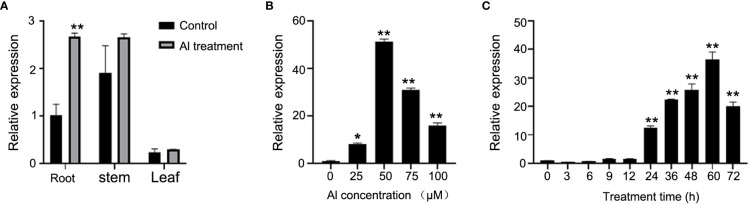
Expression pattern analysis of *GmABR1*. **(A)** Tissue expression pattern of *GmABR1*. Samples were obtained from root, stem, and leaf tissues of HX3 seedlings grown in the nutrient solutions containing 0 μM AlCl_3_ and 50 μM AlCl_3_, respectively. **(B)** Gene expression of *GmABR1* in the root of soybean cultivar HX3. The soybean cultivar HX3 was cultured for 24 h in the nutrient solutions containing 0, 25, 50, 75 and 100 μM AlCl_3_, respectively. **(C)** Time course expression pattern of *GmABR1* in the root of soybean cultivar HX3. Using soybean *Actin3* as internal reference gene, three independent replicates were detected by 2^^-△△CT^ method. The asterisks in figure **A** indicate significant differences between roots and other soybean tissues (*P < 0.05; **P < 0.01). The asterisk in figure B indicates that there is a significant difference between 0 μM AlCl_3_ and other Al concentration (25, 50, 75 and 100 μM AlCl_3_) treatment (*P < 0.05; **P < 0.01). The asterisk in figure C indicates that there is a significant difference between the Al treatment time of 0 h and the other Al treatments of 3, 6, 9, 12, 24, 36, 48, 60, 72 h (*P < 0.05; **P < 0.01).

The expression pattern of *GmABR1* was studied by using the samples of roots, stems and leaves of soybean HX3. The qRT-PCR results showed that *GmABR1* was mainly expressed in stems, followed by roots and leaves without Al stress. After Al stress, the transcripts of *GmABR1* gene was significantly increased in roots, but no significant change in stems and leaves (**Figure 3A**). In addition, the transcripts of *GmABR1* increased first, then decreased and finally increased with the increase of Al^3+^ concentration. When treated with 50 µM AlCl_3_, the transcripts of *GmABR1* were 51 times that of the control, while high concentration of AlCl_3_ inhibited the expression of *GmABR1* (**Figure 3B**). When treated with 50 µM AlCl_3_, the *GmABR1* transcripts increased gradually and then decreased along with time, and reached the highest level at 60 h after Al treatment, which was 36 times that of the control (**Figure 3C**).

### 3.4 Overexpression of *GmABR1* enhances Al resistance in transgenic Arabidopsis

**Figure 4:**
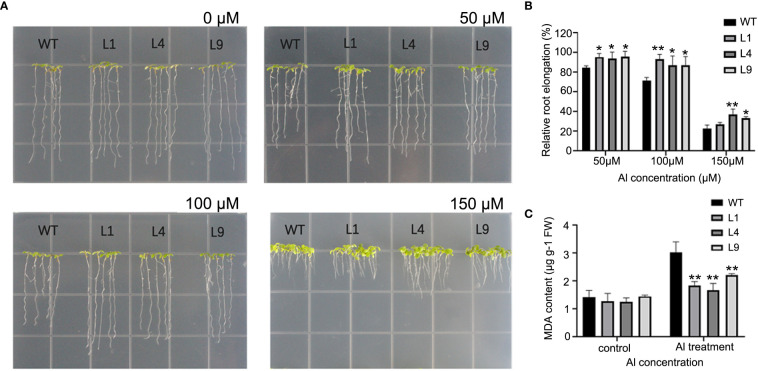
Overexpression of *GmABR1* enhances Al resistance in transgenic Arabidopsis. **(A)** Root growth of WT and *GmABR1* transgenic Arabidopsis under 0 µM, 50 µM, 100 µM, 150 µM AlCl_3_ (pH4.5). **(B)** Relative root elongation of WT and *GmABR1* transgenic Arabidopsis. **(C)** MDA content in leaves of WT and *GmABR1* transgenic Arabidopsis. The asterisk indicated that there was significant difference between WT and *GmABR1* transgenic lines (*P < 0.05; **P < 0.01). WT: Columbia-0; L1, L4, L9: *GmABR1* transgenic lines of T_3_ generation.

To investigate the effect of *GmABR1* response to Al stress, *GmABR1* was overexpressed in Arabidopsis and more than 10 homozygous lines were obtained. Three lines with high *GmABR1* transcripts (L1, L4 and L9) were selected for subsequent phenotypic identification (**Table S5**). The results showed that there was no significant difference in taproot elongation between WT and transgenic lines without Al treatments. After AlCl_3_ treatments, all lines were significantly inhibited. When AlCl_3_ concentration was 100 µM, the taproot elongation of WT was 69.6%, while that of transgenic lines was 85.6%. The taproot elongation of *GmABR1* transgenic lines was significantly higher than that of WT (**Figures 4A, B**). With the increase of AlCl_3_ concentration, the effect of inhibiting taproot elongation increased. Under the treatment of AlCl_3_, the concentration of MDA in WT was 1.41 µg/g, and that in *GmABR1* transgenic Arabidopsis was 1.32 µg/g. After 50 µM AlCl_3_ (pH 4.5) treatment, the MDA content in WT increased to 3.03 µg/g, and the MDA content in transgenic Arabidopsis leaves increased slightly up to 1.66-2.21 µg/g, which was still significantly lower than that in WT (**Figure 4C**).

### 3.5 Soybean hairy roots experiment and hematoxylin staining

**Figure 5:**
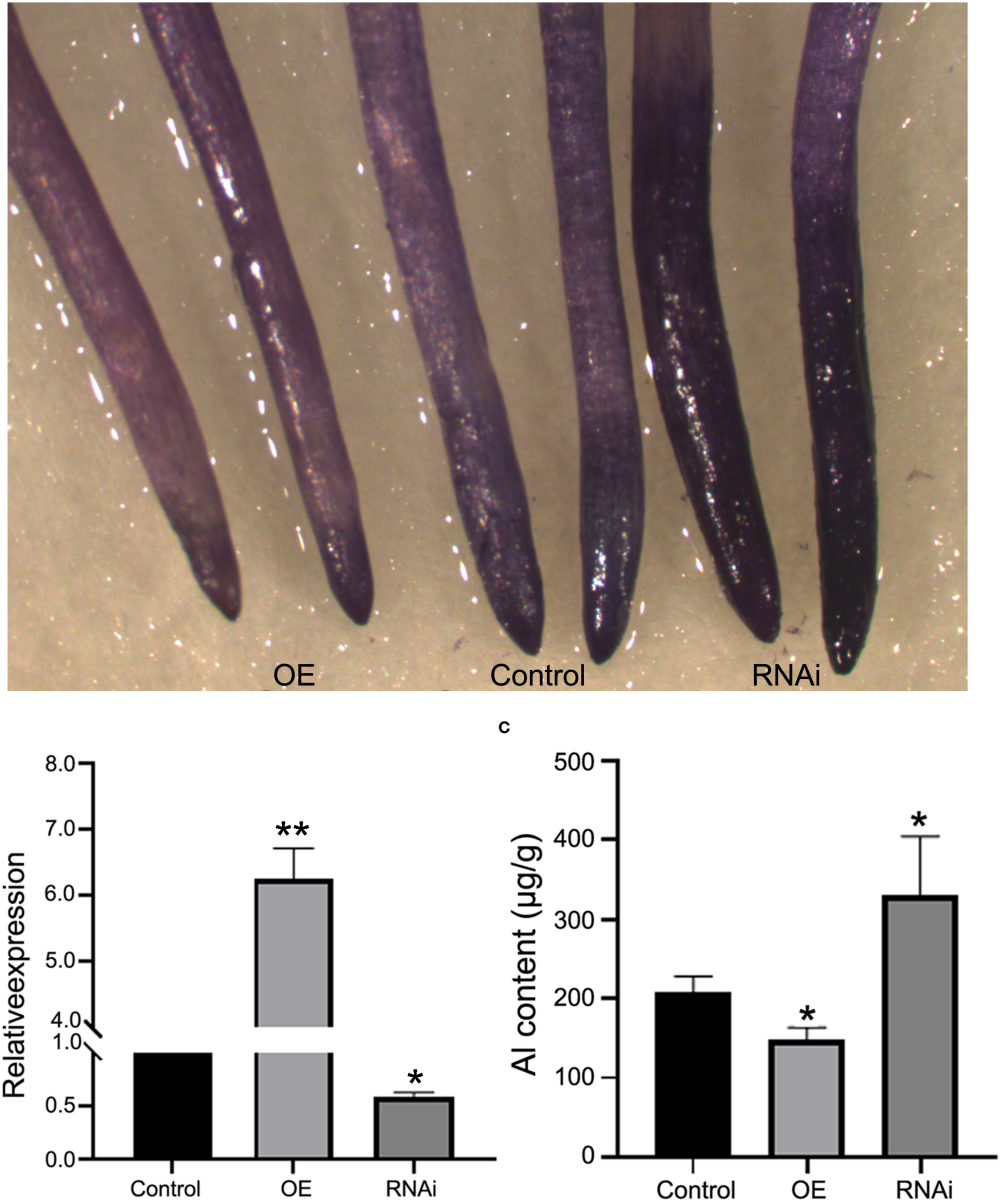
Soybean hairy roots experiment and hematoxylin staining. **(A)** Hematoxylin staining of soybean hairy roots. **(B)** Identification of RNA levels in hairy roots of soybean. **(C)** Determination of Al^3+^ content in root tips of soybean hairy root lines. The asterisks in figure B and C indicate significant differences between WT and OE or RNAi lines (*P < 0.05; **P < 0.01). Control: the lines of transformed pTF101 vector; OE: the lines of transformed pTF101-GmABR1 vector; RNAi: the lines of transformed pMU103-GmABR1 vector.

The effect of *GmABR1* gene on Al stress was studied by soybean hairy roots experiment. Soybean gene *Actin3* was used as internal reference gene, and the hair root lines transformed by pTF101 vector were used as control. The transcription level of *GmABR1* in overexpressed root lines was 6.35 times that of the control, while the transcription level of *GmABR1* in RNAi lines was only 0.60 time that of the control (**Figure 5A**). After 50 µM AlCl_3_ treatment, the results of hematoxylin staining showed that the overexpressed lines of *GmABR1* had the lightest root tip coloring, while the RNAi lines of *GmABR1* had the deepest root tip coloring (**Figure 5B**). The concentration of Al^3+^ in root tips of control, RNAi lines and overexpressed lines was 207.40 µg/g, 147.74 µg/g and 330.65 µg/g, respectively (**Figure 5C**). The accumulation of Al^3+^ in root tip of RNAi lines was significantly higher than that of *GmABR1*-overexpressed lines.

### 3.6 *GmABR1* improved the transcription level of Al stress responsive genes in Arabidopsis

**Figure 6:**
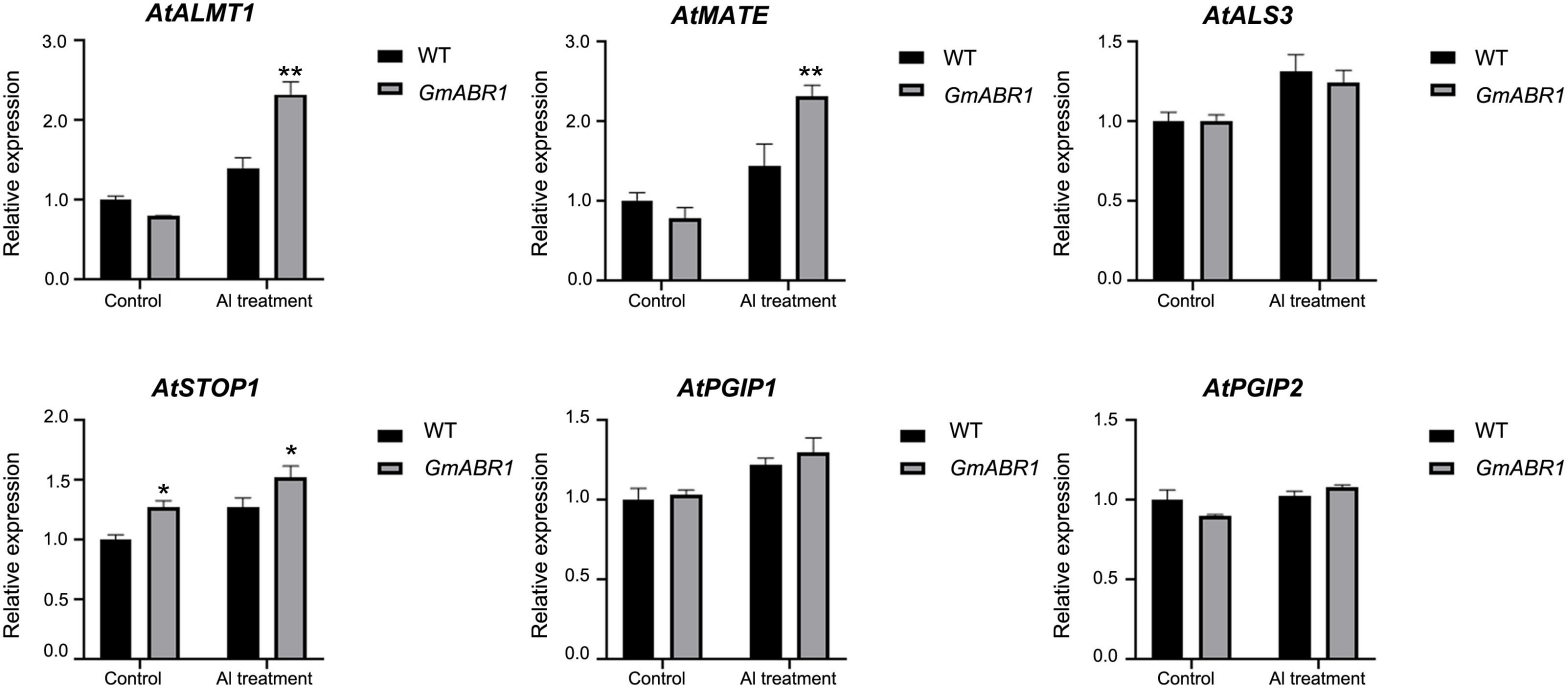
Expression patterns of Al stress responsive genes regulated by *GmABR1* in Arabidopsis. Arabidopsis were cultured in solid medium supplemented with 0 and 100 μM AlCl_3_ (pH4.5) for 7 days, respectively. The expression patterns of Al stress responsive genes by *GmABR1* were detected. Using Arabidopsis *Tubulin* as the internal reference gene, qRT-PCR was used to detect the gene expression level before and after Al treatment. The asterisks indicated that the transcripts of investigated genes in *GmABR1* transgenic lines were significantly different from those of WT treated with and without AlCl_3_ (*P < 0.05; **P < 0.01). The information of gene sequences was from the NCBI database (https://www.ncbi.nlm.nih.gov/). qRT-PCR primer information used in this experiment was shown in **Table S2**.

To further study the response mechanism of *GmABR1* to Al stress, the relative genes including *AtALMT1*, *AtMATE*, *AtALS3*, *AtSTOP1*, *AtPGIP1* and *AtPGIP2* in Arabidopsis were identified based on previous studies. The qRT-PCR analysis showed that the expression levels of some Al stress-related genes such as *AtALMT1*, *AtMATE*, *AtALS3*, *AtSTOP1*, *AtPGIP1* and *AtPGIP2* in WT and transgenic Arabidopsis were up-regulated after 100 μM AlCl_3_ (pH4.5) treatment. In particular, the expression levels of *AtALMT1* and *AtMATE* genes were significantly higher than those before aluminum treatment, which were 2.9 times and 3.0 times of those before aluminum treatment, respectively (**Figure 6**).

### 3.7 Expression changes of ABA responsive genes in Arabidopsis

**Figure 7:**
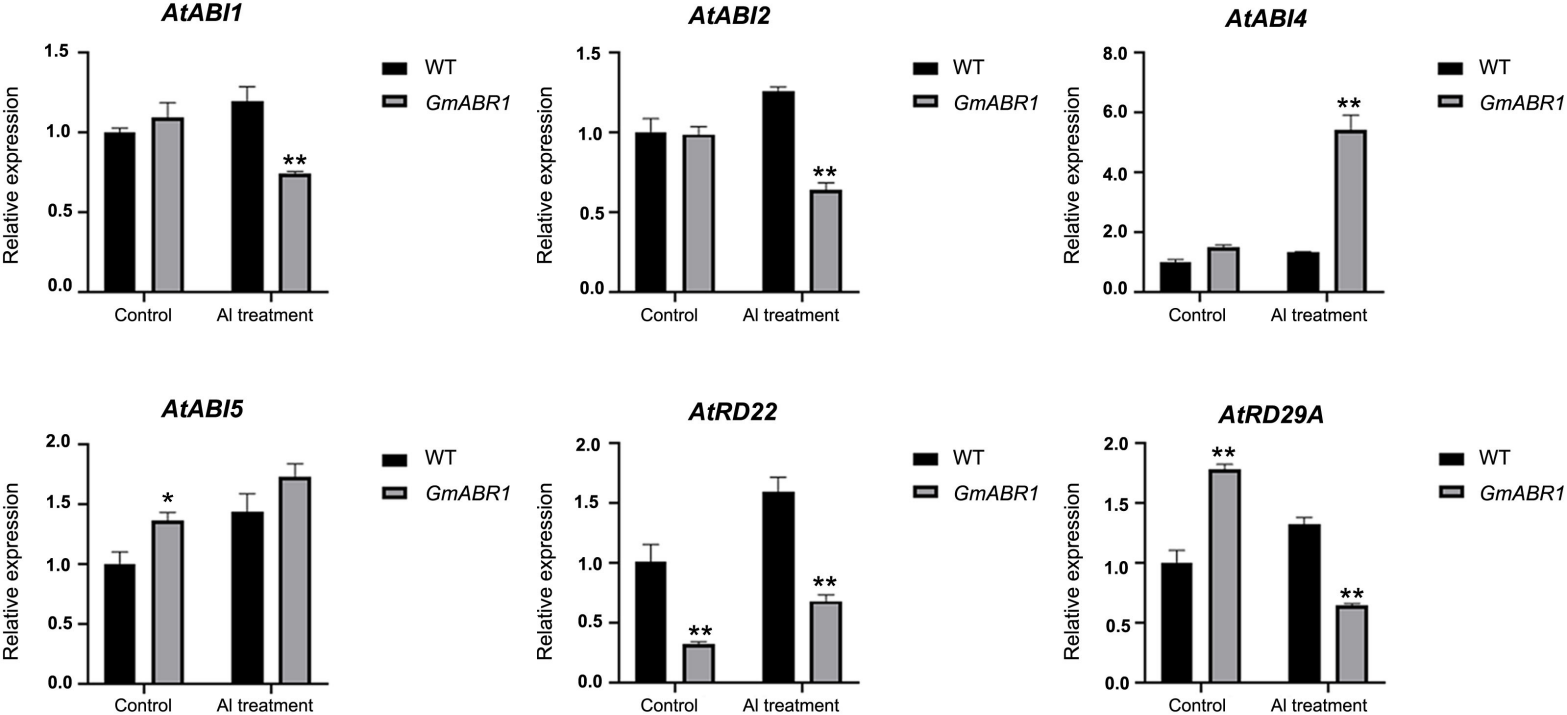
Expression patterns of ABA-responsive genes regulated by *GmABR1* in Arabidopsis. Arabidopsis were cultured in solid medium supplemented with 0 and 100 μM AlCl_3_ (pH4.5) for 7 days, respectively. The expression patterns of Al stress responsive genes by *GmABR1* were detected. Using Arabidopsis *Tubulin* as the internal reference gene, qRT-PCR was used to detect the gene expression level before and after Al treatment. The asterisks indicated that the transcripts of investigated genes in *GmABR1* transgenic lines were significantly different from those of WT treated with and without AlCl_3_ (*P < 0.05; **P < 0.01). The information of gene sequences was from the NCBI database (https://www.ncbi.nlm.nih.gov/). qRT-PCR primer information used in this experiment was shown in **Table S2**.

To further investigate the mechanism of *GmABR1* response to Al stress, the internal reference gene *Tubulin* in Arabidopsis was used as a control. The qRT-PCR analysis showed that after 100 µM AlCl_3_ (pH 4.5) treatment, the expression levels of *AtABI1*, *AtABI2*, *AtABI4*, *AtABI5*, *AtRD22* and *AtRD29A* were all up-regulated in WT. The up-regulations of *AtABI4*, *AtABI5* and *AtRD22* were 2.9, 3.0 and 2.1 times higher than those before Al stress, respectively. *AtABI1*, *AtABI2* and *AtRD29A* were down-regulated to 0.7, 0.7 and 0.4 times of the pre-treatment levels, respectively (**Figure 7**).

## 4 Discussion

Previous studies have shown that AP2/ERF family genes play important biological functions in plants including regulating plant growth and development, and coping with various abiotic stresses. Transcription factor ERF96 is a transcriptional activator that binds GCC and positively regulates Arabidopsis resistance to necrotize pathogens by responding to jasmonic acid and ethylene (Wang et al., 2015). *WRI4* which is mainly expressed in Arabidopsis stem epidermis and encodes an AP2/ERF transcription factor is up-regulated under salt stress and involved in activating epidermal wax biosynthesis to protect plants from environmental stress (Park et al., 2016). *PpeERF2* regulates peach fruit ripening by inhibiting the expression of two ABA biosynthesis genes (*PpeNCED2*, *PpeNCED3*) and cell wall degradation gene (*PpePG1*) (Wang et al., 2019b). The expression of *SlERF5* in tomato is induced not only by low temperature, injury, high salt and drought, but also by plant hormones ethylene and ABA. Transgenic plants overexpressing *SlERF5* showed increased tolerance to drought and high salinity (Wang et al., 2019b).

GmABR1 protein with a highly conserved AP2 domain had the highest homology with ERF111 (*AT5g64750*) protein in Arabidopsis. Previous studies indicated that the *AtERF111* gene was specifically induced by flooding and hypoxia. The root hair length and number of overexpressed *AtERF111* plants increased, which strengthened the hypothesis that *AtERF111* was involved in injury response (Baumler et al., 2019). *AtERF6* plays an active role in antioxidant regulation of Arabidopsis in response to biotic and abiotic stresses (Sewelam et al., 2013). In addition, GmABR1 protein is localized in the nucleus and is self-activating like other ERF transcription factors. We speculate that *GmABR1* has potential functions as a member of the ERF family as a transcription factor (Baumler et al., 2019).

In this study, Al stress can induce high expression of *GmABR1* in cultivated soybean HX3, and it is expressed in soybean roots, stems and leaves (**Figure 3**). Previous studies have shown that Fe^3+^ and Al^3+^ can form stable complexes or chelates with the components in the dye solution of hematoxylin (Hajiboland et al., 2013). In the dyeing process, oxidation film will form on the surface of the dye solution, and blue precipitation will appear in the dye solution to color the plant nucleus. The content of Al^3+^ in plant can be determined by observing the color depth. Rincon et al. exposed the roots of whole wheat seedlings to Al for 24 hours with the method of hemoxylin staining, and found that the main difference of Al accumulation between the Al tolerant (Atlas 66) and sensitive (Tam 105) varieties appeared in the growth area 0~2 and 2~5 mm from the root tip (Rincon and Gonzales, 1992). Our study showed that after hemoxylin staining of Al treated hairy roots of soybean, the root tip color of RNAi lines had the deepest color, followed by control lines, and the root tip color of overexpressed lines was stained the lightest (**Figure 5A**). Furthermore, plasma inductively coupled technique (ICP-AES) was used to determine the root tip Al^3+^ data, and the root tip Al^3+^ content of overexpressed lines was the lowest, while the root tip Al^3+^ content of RNAi lines was the highest. The results indicated that *GmABR1* can improve the ability of soybean Al detoxification (**Figure 5C**). *GmABR1* was heterologous transformed into Arabidopsis, and the relative root elongation of transgenic lines after Al treatment was significantly higher than that of WT (**Figure 4**). MDA is one of the important products of membrane lipid oxidation, and its content reflects the degree of stress damage to plant cell membrane (Luan et al., 2018). Under Al stress, MDA concentration increased significantly in WT leaves compared with transgenic plants (**Figure 4C**). These results suggest that *GmABR1* enhances the tolerance of plants to Al stress.

Previous studies have found that Al-activated secretion of malate and citric acid has important effects on plant Al tolerance. After *MATE* knockdown in Arabidopsis, the transcript level of *MATE* was reduced, and Al-activated root citrate secretion was eliminated. Moreover, *ALMT1* and *MATE* double mutants lacked Al-activated root malate and citrate. Malate secretion mediated by *ALMT1* and citrate secretion mediated by *MATE* evolved independently in Arabidopsis and together contribute to Al tolerance in Arabidopsis (Liu et al., 2009). *STOP1* encodes a zinc finger protein, which is essential for Arabidopsis to resist low pH conditions, and the involvement of *STOP1* is also required for *ALMT1* expression (Iuchi et al., 2007). Liu et al. further demonstrated that the Al tolerance function of *STOP1* also requires *MATE* expression and root citric acid secretion, Arabidopsis *STOP1* gene plays a crucial role in the Al induced expression of *MATE* and *ALMT1* (Liu et al., 2009). Recent studies have reported that *STOP1* transcription is not affected by Al stress, but the expression of Al resistance genes *ALMT1*, *MATE* and *ALS3* downstream of *STOP1* is induced by Al stress. These results suggest that *STOP1* may be regulated by Al at the post-transcriptional or post-translational level (Zhang et al., 2019b). *ALS3* is predicted to encode a factor required for root growth in an Al-toxic environment, and mutational loss of this factor leads to severe Al-dependent consequences. This factor is specific for Al tolerance, as *ALS3* loss-of-function mutants do not show increased sensitivity to other metals, including La and Cu, nor do they exhibit any abnormal growth phenotype in the absence of Al (Larsen et al., 1997). Yuriko et al. found that *PGIP1* and *PGIP2* were strongly inhibited in *STOP1* mutants under Al stress, *PGIP1* and *PGIP2* may play a regulatory role in Al tolerance (Kobayashi et al., 2014). The Al-sensitive gene *ALS3* encodes an ABC transporter-like protein that is required for resistance tolerance to Al to redistribute accumulated Al from sensitive tissues, thereby protecting growing roots from Al toxicity (Larsen et al., 2005).

In present study, the expression levels of *AtALMT1*, *AtMATE*, *AtALS3*, *AtSTOP1*, *AtPGIP1* and *AtPGIP2* genes were up-regulated in WT and transgenic Arabidopsis. The transcription levels of *AtALMT1* and *AtMATE* in transgenic Arabidopsis were significantly increased compared with those before AlCl_3_ treatment (**Figure 6**). Therefore, the results indicated that *GmABR1* may increase the secretion of malate and citrate in root tips of transgenic Arabidopsis by inducing the expression of Al stress response genes *AtALMT1* and *AtMATE*, and then increase the resistance of transgenic Arabidopsis to Al stress through external Al exclusion mechanism.

ABA is an important endogenous messenger for plants to cope with stress. Yang et al. found that *AtERF4* is a negative regulator of ethylene and abscisic acid response (Yang et al., 2005). Zhu et al. found that overexpression of *RAP2.6*, a member of the AP2/ERF family, could induce hypersensitivity responses to exogenous ABA and abiotic stress during seed germination and early seedling growth of Arabidopsis. ABA content in *RAP2.6* overexpressing lines decreased after salt treatment (Zhu et al., 2010). The *AtRAP2.6L* homologous apple gene *MdERF113* positively regulates abiotic stress and ABA stress (Rui et al., 2023). In recent years, Arabidopsis TCTP transcription factors have been gradually proved to be relative to abiotic stress signals. Overexpression of *AtTCTP* can enhance drought tolerance of plants by inducing ABA-mediated stomatal closure through interaction with microtubules (Kim et al., 2012). Merlot et al. found that double mutant plants of *ABI1* and *ABI2* loss function alleles were more responsive to ABA than their parental single mutants, while *ABI1* and *ABI2* were jointly involved in the negative feedback regulation of ABA signaling pathway (Merlot et al., 2001). ABI4 is also an important transcription factor in ABA signaling pathway, which can positively regulate *NCED6*, a key gene in ABA biosynthesis (Shu et al., 2016). Some studies have shown that Arabidopsis *ABI5* mutants have enhanced sensitivity to Al, however, it is not related to the regulation of *ALMT1* and *MATE* expression, indicating that ABA signal transduction pathway provides an additional regulatory mechanism for plant Al tolerance. The positive feedback regulatory transcription factor ABI5 can mediate cell wall modification and osmoregulation to enhance plant Al tolerance (Fan et al., 2019). The *RD29A* and *RD22* genes are very sensitive to various abiotic stressors, and their expression is induced by endogenous ABA, which can enhance the ability of plants to resist abiotic stress by participating in ABA pathway (Yamaguchi-Shinozaki and Shinozaki, 1993; Lee et al., 2016). *AtALMT12* is involved in ABA-induced stomatal closure and is a hypothesized target of this pathway with high nitrate and chloride transport capacity, stomatal closure may confer resistance to aluminum (Sasaki et al., 2010). Regulation of stomatal conductance by ABA signals may be an effective strategy to enhance abiotic stress in plants (Hsu et al., 2021).

In this study, the expression levels of *AtABI1*, *AtABI2*, *AtABI4*, *AtABI5*, *AtRD22* and *AtRD29A* genes in WT were all up-regulated after Al treatment. Among them, the transcripts of *AtABI4*, *AtABI5* and *AtRD22* in transgenic lines were significantly up-regulated under Al stress. While the expressions of *AtABI1*, *AtABI2* and *AtRD29A* were down-regulated with lower transcripts of the control treatment (**Figure 7**). The *RD29A* gene was up-regulated by Al stress in WT plants, but decreased in the transgenic lines. We hypothesized that this pathway has a compensating effect for the acid-resistant Al phenotype of the transgenic lines. Msanne et al. also found contrary to expectations that *RD29A* knockout mutant lines maintained greater root growth, photosynthesis and water use efficiency under salt stress than those of the controls. All transgenes effectively complemented resistance to salt stress-induced root growth inhibition (Msanne et al., 2011). Therefore, the results indicated that *GmABR1* maybe also enhance plant resistance to abiotic stress by participating in the ABA pathway (Xu et al., 2017; Huang et al., 2022).

In summary, the *GmABR1* gene encoding a AP2/ERF transcription factor in soybean was identified in present study, which was induced by Al stress and enriched in the roots, leaves, stems of soybean seedlings. *GmABR1* overexpression increased the tolerance of *GmABR1* transgenic plants to Al stress. The analysis of molecular mechanism showed that the enhanced tolerance to Al stress might due to the comprehensive roles in upregulating and/or downregulating transcripts of the Al stress- and/or ABA-responsive genes with MDA decrease. Therefore, these results suggested that *GmABR1* may jointly regulate plant resistance to Al stress through the genes related to Al response and/or ABA response pathways.

## Data availability statement

The data presented in the study are deposited in the NCBI database (https://www.ncbi.nlm.nih.gov/). The entry numbers of all genes involved in this paper (including the genetic information for making evolutionary trees) can be found in **Supplementary Material Table S6**. The original contributions presented in the study are included in the article/**Supplementary Material**, further inquiries can be directed to the corresponding author/s.

## Author contributions

QM, AZ and HW conceived of and designed the study. HW, CL, LW, HZ, XX, YC, WL and HN conducted the experiments. HW, CL, LW, PC and QM performed data as well as statistical analysis. HW prepared the manuscript which was edited by QM and AZ. All authors contributed to the article and approved the submitted version.
