# Supplementary material for: GmABR1 encoding an ERF transcription factor enhances the tolerance to aluminum stress in Arabidopsis thaliana
Source: Front Plant Sci. 2023 Mar 23;14:1125245. doi: 10.3389/fpls.2023.1125245 (PMC10076715; doi:10.3389/fpls.2023.1125245)
Supplement: Supplementary file 1 [file DataSheet_1.zip › Raw Data/GmABR1-Table S1-S5.docx]

Supplementary Material

**Table S1.** **Sequence information of *GmABR1***

**Transcript sequence information of *GmABR1***

AGATTGAAGGGGTAGTATTTTTGCTTTTGCATGCATCAATCAGAAACGAGGGATTCGATCTAGTAGCTAACTAAAACAAGCTTTTTGATTGCGTTTACACGTGTTTGTTAAAGGTGGCAAATCCTAAAGAGAAGAAGGGAAACGAGGTTGACAATATATTATTCCATGAAGGGATGATGAACAACCCTGCATTATTGTCAAGGCTGTTTCCGGGTATCAATAGGGAGAGGGAGATGTCTGCCATGGTCTCAGCTTTGACCCACGTTGTCTCCGGAGAGGTTCCAACCGGTGATTCTTCTTCAGTTCTTCATCATCAACCTCATGATGATGATGCTGTTACTGTTAAACCAAACATGCCTTCTACAACACCCTTTTCATCTACTTATGTTGTTGCCACCTCTTCTCTCAAGAGAAGTAGACAACATGATACATATACACCATCCTTCTCACATCCACCTTCATCATTCCCTGCTATGCCAACAAGTGCAGAATGTTCAAAGAGTGGAATGGGAAATGCTGTGTACGAATACAGAACAAGAGGAACAGAGAATGTAAAGAAGGAGGAGGGAAGGAGAAAATATAGAGGGGTGAGGCAGAGGCCGTGGGGAAAATGGGCAGCGGAGATAAGAGACCCATTCAAAGCAGCAAGAGTATGGTTAGGAACATTTGAGACGGCAGAGGCAGCAGCTAGAGCCTACGATGAAGCTGCCCTTCGTTTTAGGGGCAGCAAGGCCAAACTCAACTTCCCCGAGAATGTCACTCTCCGACAACCTCAATTCACTGTTTCCACATCAACAGACCCATTTCAACTCTCGGGTACCAACCTTTATGATCATAGGATGTTTCTGACTTCTAATACCATGGCTTCTCATCATCTTCAATCTTCTTTGCCGCTGCCACCAGCAACCTCCTCTTCTCTAACTTCTCCTCCTCAGAACATCACTTCACTTTCTTCCTTGTATTCTGCCCAGTTGCCCCCATGGTCGGCCTCTGGTCATAGCTCTTCTTCCTCCCCATGATCCTTTTTTGTTTCTTTGTTTTTATTGAGTCTATATTTTCCCGTCTTTTATCGTATCTCTCCATTTTTTTCAAACACTAATTAATAATAATAATAATAATTATTCCTCTGTTATTG

**CDS sequencing** **information of *GsMYB7***

ATGATGAACAACCCTGCATTATTGTCAAGGCTGTTTCCGGGTATCAATAGGGAGAGGGAGATGTCTGCCATGGTCTCAGCTTTGACCCACGTTGTCTCCGGAGAGGTTCCAACCGGTGATTCTTCTTCAGTTCTTCATCATCAACCTCATGATGATGATGCTGTTACTGTTAAACCAAACATGCCTTCTACAACACCCTTTTCATCTACTTATGTTGTTGCCACCTCTTCTCTCAAGAGAAGTAGACAACATGATACATATACACCATCCTTCTCACATCCACCTTCATCATTCCCTGCTATGCCAACAAGTGCAGAATGTTCAAAGAGTGGAATGGGAAATGCTGTGTACGAATACAGAACAAGAGGAACAGAGAATGTAAAGAAGGAGGAGGGAAGGAGAAAATATAGAGGGGTGAGGCAGAGGCCGTGGGGAAAATGGGCAGCGGAGATAAGAGACCCATTCAAAGCAGCAAGAGTATGGTTAGGAACATTTGAGACGGCAGAGGCAGCAGCTAGAGCCTACGATGAAGCTGCCCTTCGTTTTAGGGGCAGCAAGGCCAAACTCAACTTCCCCGAGAATGTCACTCTCCGACAACCTCAATTCACTGTTTCCACATCAACAGACCCATTTCAACTCTCGGGTACCAACCTTTATGATCATAGGATGTTTCTGACTTCTAATACCATGGCTTCTCATCATCTTCAATCTTCTTTGCCGCTGCCACCAGCAACCTCCTCTTCTCTAACTTCTCCTCCTCAGAACATCACTTCACTTTCTTCCTTGTATTCTGCCCAGTTGCCCCCATGGTCGGCCTCTGGTCATAGCTCTTCTTCCTCCCCATGA

**Amino acid sequence of GmABR1 protein**

MMNNPALLSRLFPGINREREMSAMVSALTHVVSGEVPTGDSSSVLHHQPHDDDAVTVKPNMPSTTPFSSTYVVATSSLKRSRQHDTYTPSFSHPPSSFPAMPTSAECSKSGMGNAVYEYRTRGTENVKKEEGRRKYRGVRQRPWGKWAAEIRDPFKAARVWLGTFETAEAAARAYDEAALRFRGSKAKLNFPENVTLRQPQFTVSTSTDPFQLSGTNLYDHRMFLTSNTMASHHLQSSLPLPPATSSSLTSPPQNITSLSSLYSAQLPPWSASGHSSSSSP*

**Insert sequences in RNAi**

ACTTCTAATACCATGGCTTCTCATCATCTTCAATCTTCTTTGCCGCTGCCACCAGCAACCTCCTCTTCTCTAACTTCTCCTCCTCAGAACATCACTTCACTTTCTTCCTTGTATTCTGCCCAGTTGCCCCCATGGTCGGCCTCTGGTCATAGCTCTTCT

**Table S2. The primers used in the study**

| **Primer Name** | **Purpose** | **Forward Primer (5'-3')** |
| --- | --- | --- |
| GmABR1-F | [Gene clone](javascript:;) | aagaagggaaacgaggttgaca |
| GmABR1-R | [Gene clone](javascript:;) | gatacgataaaagacgggaaaa |
| P1302-ABR1-F | Recombinant clone | acgggggactcttgaccatggCTATGATGAACAACCCTGCATTATTG |
| P1302-ABR1-R | Recombinant clone | aagttcttctcctttactagtTGGGGAGGAAGAAGAGCTATGA |
| pTF-ABR1-F | Recombinant clone | gaacacgcaggggactctagaatgatgaacaaccctgcattattg |
| pTF-ABR1-R | Recombinant clone | atcggggaaattcgtgagctctcatggggaggaagaagagct |
| pMU-ABR1-1F | Recombinant clone | ctttttgtatgtaccggcgcgccACTTCTAATACCATGGCTTCTCATCA |
| pMU-ABR1-1R | Recombinant clone | atatatgtataccttcctaggAGAAGAGCTATGACCAGAGGCCG |
| pMU-ABR1-2F | Recombinant clone | caggactctagacccactagtACTTCTAATACCATGGCTTCTCATCA |
| pMU-ABR1-2R | Recombinant clone | caattcaattcagtggagctcAGAAGAGCTATGACCAGAGGCCG |
| P1302-F | Identification of monoclones | TGTGAAGATAGTGGAAAAG |
| P1302-R | Identification of monoclones | GCCACGGAACAGGTAGTTT |
| pTF101-F | Identification of monoclones | CCTTCGCAAGACCCTTCCTC |
| pTF101-R | Identification of monoclones | TCATCGCAAGACCGGCAAC |
| ABI1-F | qRT-PCR | AGAGTGTGCCTTTGTATGGTTTTA |
| ABI1-R | qRT-PCR | CATCCTCTCTCTACAATAGTTCGCT |
| ABI2-F | qRT-PCR | GATGGAAGATTCTGTCTCAACGATT |
| ABI2-R | qRT-PCR | GTTTCTCCTTCACTATCTCCTCCG |
| ABI4-F | qRT-PCR | ACTCCAAGTTCCGTTACCGTG |
| ABI4-R | qRT-PCR | GGGGTTAAGTTGAGCTGAGCA |
| ABI5-F | qRT-PCR | CAATAAGAGAGGGATAGCGAACGAG |
| ABI5-R | qRT-PCR | CGTCCATTGCTGTCTCCTCCA |
| RD29A-F | qRT-PCR | GGCGTAACAGGTAAACCTAGAG |
| RD29A-R | qRT-PCR | TCCGATGTAAACGTCGTCC |
| RD22-F | qRT-PCR | GGTTCGGAAGAAGCGGAG |
| RD22-R | qRT-PCR | GAAACAGCCCTGACGTGATAT |
| ALMT1-F | qRT-PCR | GGCAGTGTGCCTACAGGATT |
| ALMT1-R | qRT-PCR | TGAGTTTCCCGATTCCGAGC |
| ALS3-F | qRT-PCR | CAATCGCCGGAATGTTGGTC |
| ALS3-R | qRT-PCR | TTGCAACGTCGCTTGTCTTG |
| MATE-F | qRT-PCR | GTAGCTGGCCAGGCAATACT |
| MATE-R | qRT-PCR | AACTGCAGTACACGAGAGGC |
| STOP1-F | qRT-PCR | ACGGTTGAGATTAATGGGTTGAC |
| STOP1-R | qRT-PCR | CTGAGCGGGGCTCATGTTTA |
| PGIP1-F | qRT-PCR | ACTGTTGCTCCTGGTACTGC |
| PGIP1-R | qRT-PCR | GCGGAAGACAAGGGTCTCAA |
| PGIP2-F | qRT-PCR | TCAACCACCGTGTCACTTCC |
| PGIP2-R | qRT-PCR | TGGCGATAGTGGGTTGGATG |
| Actin3-F | qRT-PCR | GCACCACCGGAGAGAAAATA |
| Actin3-R | qRT-PCR | GTGCACAATTGATGGACCAG |
| Tubulin-F | qRT-PCR | ATCGATTCCGTTCTCGATGT |
| Tubulin-R | qRT-PCR | ATCCAGTTCCTCCTCCCAAC |
| qABR1-F | qRT-PCR | AGCTGCCCTTCGTTTTAGGG |
| qABR1-R | qRT-PCR | GACCCATTTCAACTCTCGGGT |

**Table S3.** **Gene information for protein sequence alignment**

| **Serial number** | **Gene**  **name** | **Protein sequence number** | **References** |
| --- | --- | --- | --- |
| 1 | *AtERF111* | [NP_201280.1](https://www.ncbi.nlm.nih.gov/protein/NP_201280.1) | Pandey G K, Grant J J, Cheong Y H, *et al*. *ABR1*, an APETALA2-domain transcription factor that functions as a repressor of ABA response in Arabidopsis [J]. Plant Physiology, 2005, 139(3): 1185-1193. |
| 2 | *AtERF11* | NP_001319098.1 | Zhou X, Zhang Z L, Park J, *et al*. The *ERF11* transcription factor promotes internode elongation by activating gibberellin biosynthesis and signaling [J]. Plant Physiology, 2016, 171(4): 2760-2770. |
| 3 | *AtERF4* | [NP_188139.1](https://www.ncbi.nlm.nih.gov/protein/NP_188139.1) | Liu W, Karemera N, Wu T, *et al*. The ethylene response factor *AtERF4* negatively regulates the iron deficiency response in Arabidopsis thaliana [J]. PLoS One, 2017, 12(10): e186580. |
| 4 | *AtERF8* | [NP_568679.1](https://www.ncbi.nlm.nih.gov/protein/NP_568679.1) | Moffat C S, Ingle R A, Wathugala D L, *et al*. *ERF5* and *ERF6* play redundant roles as positive regulators of JA/Et-mediated defense against Botrytis cinerea in Arabidopsis[J]. PLoS One, 2012, 7(4): e35995. |
| 5 | *AtERF9* | [NP_199234.1](https://www.ncbi.nlm.nih.gov/protein/NP_199234.1) | Maruyama Y, Yamoto N, Suzuki Y, *et al*. The Arabidopsis transcriptional repressor *ERF9* participates in resistance against necrotrophic fungi [J]. Plant Science, 2013, 213: 79-87. |
| 6 | *AtERF12* | [NP_174158.1](https://www.ncbi.nlm.nih.gov/protein/NP_174158.1) | Chandler J W, Werr W. A phylogenetically conserved APETALA2/ethylene response factor, *ERF12*, regulates Arabidopsis floral development [J]. Plant Molecular Biology, 2020, 102(1-2): 39-54. |
| 7 | *AtERF71* | [NP_182274.1](https://www.ncbi.nlm.nih.gov/protein/NP_182274.1) | Lee S Y, Hwang E Y, Seok H Y, *et al*. Arabidopsis *AtERF71/HRE2* functions as transcriptional activator via cis-acting GCC box or DRE/CRT element and is involved in root development through regulation of root cell expansion [J]. Plant Cell Reports, 2015, 34(2): 223-231. |
| 8 | *AtORA59* | [NP_172106.1](https://www.ncbi.nlm.nih.gov/protein/NP_172106.1) | Li J, Zhang K, Meng Y, *et al*. Jasmonic acid/ethylene signaling coordinates hydroxycinnamic acid amides biosynthesis through *ORA59* transcription factor [J]. Plant Journal, 2018, 95(3): 444-457. |
| 9 | *AtERF15* | [NP_850162.1](https://www.ncbi.nlm.nih.gov/protein/NP_850162.1) | Lee S B, Lee S J, Kim S Y. *AtERF15* is a positive regulator of ABA response [J]. Plant Cell Reports, 2015, 34(1): 71-81. |
| 10 | *AtERF1* | [NP_188965.1](https://www.ncbi.nlm.nih.gov/protein/NP_188965.1) | Mao J L, Miao Z Q, Wang Z, *et al*. Arabidopsis *ERF1* mediates cross-talk between ethylene and auxin biosynthesis during primary root elongation by regulating *ASA1* expression [J]. PLoS Genetics, 2016, 12(1): e1005760. |
| 11 | *AtERF14* | [NP_171932.1](https://www.ncbi.nlm.nih.gov/protein/NP_171932.1) | Onate-Sanchez L, Anderson J P, Young J, *et al*. *AtERF14*, a member of the ERF family of transcription factors, plays a nonredundant role in plant defense [J]. Plant Physiology, 2007, 143(1): 400-409. |
| 12 | *AtERF6* | [NP_567529.1](https://www.ncbi.nlm.nih.gov/protein/NP_567529.1) | Warmerdam S, Sterken M G, Van Schaik C, *et al*. Mediator of tolerance to abiotic stress *ERF6* regulates susceptibility of Arabidopsis to Meloidogyne incognita[J]. Molecular Plant Pathology, 2019, 20(1): 137-152. |
| 13 | *AtERF5* | [NP_568679.1](https://www.ncbi.nlm.nih.gov/protein/NP_568679.1) | Moffat C S, Ingle R A, Wathugala D L, *et al*. *ERF5* and *ERF6* play redundant roles as positive regulators of JA/Et-mediated defense against Botrytis cinerea in Arabidopsis[J]. PLoS One, 2012, 7(4): e35995. |
| 14 | *ATERF38* | [NP_181113.1](https://www.ncbi.nlm.nih.gov/protein/NP_181113.1) | Lasserre E, Jobet E, Llauro C, *et al*. *AtERF38* (*At2g35700*), an AP2/ERF family transcription factor gene from Arabidopsis thaliana, is expressed in specific cell types of roots, stems and seeds that undergo suberization [J]. Plant Physiol Biochem, 2008, 46(12): 1051-1061. |
| 15 | *AtERF53* | [NP_179685.1](https://www.ncbi.nlm.nih.gov/protein/NP_179685.1) | Cheng M C, Hsieh E J, Chen J H, *et al*. Correction. Arabidopsis *RGLG2*, Functioning as a RING E3 Ligase, Interacts with *AtERF53* and Negatively Regulates the Plant Drought Stress Response [J]. Plant Physiology, 2016, 170(2): 1162-1163. |
| 16 | *GmERF3* | [NP_001238300.2](https://www.ncbi.nlm.nih.gov/protein/NP_001238300.2) | Zhang G, Chen M, Li L, *et al*. Overexpression of the soybean *GmERF3* gene, an AP2/ERF type transcription factor for increased tolerances to salt, drought, and diseases in transgenic tobacco [J]. Journal of Experimental Botany, 2009, 60(13): 3781-3796. |
| 17 | *GmERN* | [XP_006604239.1](https://www.ncbi.nlm.nih.gov/protein/XP_006604239.1) | Cerri M R, Frances L, Kelner A, *et al*. The Symbiosis-related ERN transcription factors act in concert to coordinate rhizobial host root infection [J]. Plant Physiology, 2016, 171(2): 1037-1054. |
| 18 | *GmRAV1* | [NP_001358046.1](https://www.ncbi.nlm.nih.gov/protein/NP_001358046.1) | Zhang K, Zhao L, Yang X, *et al*. *GmRAV1* regulates regeneration of roots and adventitious buds by the cytokinin signaling pathway in Arabidopsis and soybean [J]. Physiol Plant, 2019, 165(4): 814-829. |
| 19 | *OsERF71* | [XP_015643752.1](https://www.ncbi.nlm.nih.gov/protein/XP_015643752.1) | Li J, Guo X, Zhang M, *et al*. O*sERF71* confers drought tolerance via modulating ABA signaling and proline biosynthesis [J]. Plant Science, 2018, 270: 131-139. |
| 20 | *OsLG3* | [XP_015630585.1](https://www.ncbi.nlm.nih.gov/protein/XP_015630585.1) | Xiong H, Yu J, Miao J, *et al*. Natural Variation in *OsLG3* Increases drought tolerance in rice by inducing ros scavenging [J]. Plant Physiology, 2018, 178(1): 451-467. |
| 21 | *OsERF073* | [XP_015632219.1](https://www.ncbi.nlm.nih.gov/protein/XP_015632219.1) | Kikuchi S, Satoh K, Nagata T, *et al*. Collection, mapping, and annotation of over 28,000 cDNA clones from japonica rice [J]. Science, 2003, 301(5631): 376-379. |
| 22 | *OsERF48* | [XP_015650910.1](https://www.ncbi.nlm.nih.gov/protein/XP_015650910.1) | Jung H, Chung P J, Park S H, *et al*. Overexpression of *OsERF48* causes regulation of *OsCML16*, a calmodulin-like protein gene that enhances root growth and drought tolerance [J]. Plant Biotechnology Journal, 2017, 15(10): 1295-1308. |

**Table S4. The target gene *GmABR1* was identified by PCR**

**
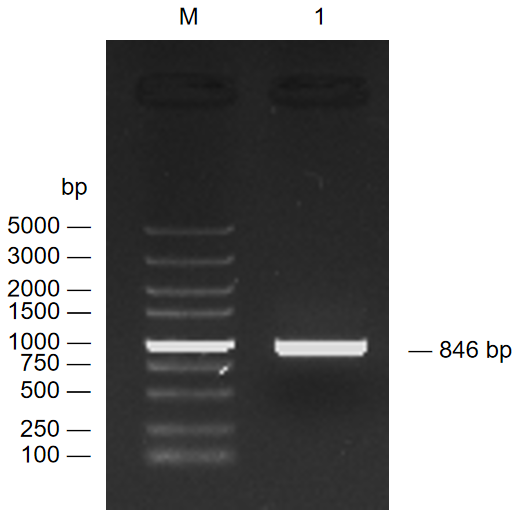
**

M：DL5000 marker； 1: PCR product of *GmABR1*.

**Table S5.** **Identification of a positive** **transgenic *Arabidopsis thaliana* lines**

**
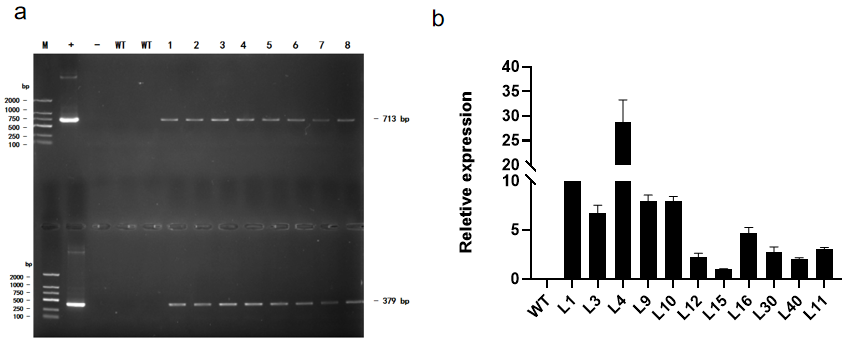
**

Positive transgenic *Arabidopsis thaliana* lines were identified by PCR using two pairs of primers. M: DL2000 marker； +:pTF101-GmABR1 plasmid; -: ddH_2_O; WT: wild-type *Arabidopsis thaliana*; 1, 2, 3, 4, 5, 6, 7, 8: transgenic Arabidopsis thaliana line. (b) Transgenic T_3_ generation *Arabidopsis thaliana* lines were detected by qRT-PCR at RNA level. The inner reference gene *Tubulin* in *Arabidopsis thaliana* was used to normalize the data. The expression data were calculated using the comparative cycle threshold method 2^-△△ct^.
