# Supplementary material for: GmABR1 encoding an ERF transcription factor enhances the tolerance to aluminum stress in Arabidopsis thaliana
Source: Front Plant Sci. 2023 Mar 23;14:1125245. doi: 10.3389/fpls.2023.1125245 (PMC10076715; doi:10.3389/fpls.2023.1125245)
Supplement: Supplementary file 1 [file DataSheet_1.zip › Raw Data/GmABR1-Table S6.docx]

**Table S6. The information of *GmABR1* genes from Arabidopsis and soybean**

| **Serial number** | **Gene** | **Gene ID** | **Descriptions** | **References** |
| --- | --- | --- | --- | --- |
| 1 | *Glyma.02G066200* | 100796480 |  |  |
| 2 | *Glyma.16G047600* | 100816370 |  |  |
| 3 | *Glyma.04G217400* | 100793215 |  |  |
| 4 | *Glyma.09G041500* | 100817253 | Enhances resistance to Phytophthora sojae | Dong, L., Cheng, Y., Wu, J., Cheng, Q., and Li, W., et al. (2015). Overexpression of GmERF5, a new member of the soybean EAR motif-containing ERF transcription factor, enhances resistance to Phytophthora sojae in soybean. J Exp Bot 66, 2635-2647. doi:10.1093/jxb/erv078 |
| 5 | *Glyma.10G036300* | 100782036 |  |  |
| 6 | *Glyma.19G163900* | 100800600 |  |  |
| 7 | *Glyma.09G052900* | 100500521 |  |  |
| 8 | *Glyma.09G052800* | 100801981 |  |  |
| 9 | *Glyma.11G036400* | 100800788 |  |  |
| 10 | *Glyma.20G155100* | 100101894 |  |  |
| 11 | *Glyma.17G131900* | 100779318 |  |  |
| 12 | *Glyma.17G131800* | 100778793 |  |  |
| 13 | *Glyma.12G182200* | 100815039 |  |  |
| 14 | *Glyma.12G182400* | 100816109 |  |  |
| 15 | *Glyma.01G074200* | 100792964 |  |  |
| 16 | *Glyma.11G035100* | 100817503 |  |  |
| 17 | *Glyma.04G057700* | 100808434 |  |  |
| 18 | *Glyma.14G106200* | 112999363 |  |  |
| 19 | *Glyma.13G112400* | 100804384 |  |  |
| 20 | *Glyma.04G041200* | 100801528 |  |  |
| 21 | *Glyma.14G084700* | 547642 |  |  |
| 22 | *Glyma.06G221800* | 100819868 |  |  |
| 23 | *Glyma.14G111600* | 100807612 |  |  |
| 24 | *Glyma.12G197700* | 100527471 |  |  |
| 25 | *Glyma.07G156200* | 109244108 |  |  |
| 26 | *Glyma.04G028100* | 100790057 |  |  |
| 27 | *Glyma.13G088100* | 100802961 |  |  |
| 28 | *Glyma.05G179900* | 100797648 |  |  |
| 29 | *Glyma.18G018200* | 106796800 |  |  |
| 30 | *Glyma.03G094700* | 100815537 |  |  |
| 31 | *Glyma.16G079600* | 100779645 |  |  |
| 32 | *Glyma.11G019000* | 100779905 |  |  |
| 33 | *Glyma.08G320700* | 100795762 |  |  |
| 34 | *Glyma.18G091600* | 100782025 |  |  |
| 35 | *Glyma.15G077100* | 100803173 |  |  |
| 36 | *Glyma.02G067600* | 100815668 |  |  |
| 37 | *Glyma.16G046300* | 100792421 |  |  |
| 38 | *Glyma.01G025400* | 102669715 |  |  |
| 39 | *Glyma.08G281900* | 102665767 | Candidate genes for controlling single leaf number traits | Jeong, S.C., Kim, J.H., and Bae, D.N. (2017). Genetic analysis of the Lf1 gene that controls leaflet number in soybean. Theoretical and Applied Genetics 130, 1685-1692. doi:10.1007/s00122-017-2918-0 |
| 40 | *Glyma.18G144700* | 102661952 |  |  |
| 41 | *Glyma.03G111700* | 100793366 |  |  |
| 42 | *Glyma.07G114300* | 100804762 |  |  |
| 43 | *Glyma.11G036500* | 100819649 |  |  |
| 44 | *AT5G64750* | 836596 | Response to submergence and hypoxia stresses | Baumler, J., Riber, W., Klecker, M., Muller, L., and Dissmeyer, N., et al. (2019). AtERF#111/ABR1 is a transcriptional activator involved in the wounding response. Plant J 100, 969-990. doi:10.1111/tpj.14490 |
| 45 | *[AT1G06160](https://bar.utoronto.ca/thalemine/portal.do?externalids=AT1G06160)* | 837125 | Regulates the plant's defense against necrotizing pathogens | Yang, Y.N., Kim, Y., Kim, H., Kim, S.J., and Cho, K.M., et al. (2021). The transcription factor ORA59 exhibits dual DNA binding specificity that differentially regulates ethylene- and jasmonic acid-induced genes in plant immunity. Plant Physiol 187, 2763-2784. doi:10.1093/plphys/kiab43 |
| 46 | *AT2G31230* | 817680 | Positive regulation of ABA and stress response | Lee, S.B., Lee, S.J., and Kim, S.Y. (2015). AtERF15 is a positive regulator of ABA response. Plant Cell Rep 34, 71-81. doi:10.1007/s00299-014-1688-2 |
| 47 | *[AT4G17490](https://bar.utoronto.ca/thalemine/portal.do?externalids=AT4G17490)* | 827463 | Play the role of oxidative stress | Sewelam, N., Kazan, K., Thomas-Hall, S.R., Kidd, B.N., and Manners, J.M., et al. (2013). Ethylene response factor 6 is a regulator of reactive oxygen species signaling in Arabidopsis. PLoS One 8, e70289. doi:10.1371/journal.pone.0070289 |
| 48 | *[AT4G25470](https://bar.utoronto.ca/thalemine/portal.do?externalids=AT4G25470)* | 828651 | Candidate genes for quantitative trait loci of cold tolerance | Alonso-Blanco, C., Gomez-Mena, C., Llorente, F., Koornneef, M., and Salinas, J., et al. (2005). Genetic and molecular analyses of natural variation indicate CBF2 as a candidate gene for underlying a freezing tolerance quantitative trait locus in Arabidopsis. Plant Physiol 139, 1304-1312. doi:10.1104/pp.105.068510 |
| 49 | *AT4G25480* | 828652 | Improve drought resistance | Wei, T., Deng, K., Liu, D., Gao, Y., and Liu, Y., et al. (2016). Ectopic expression of DREB transcription factor, AtDREB1A, confers tolerance to drought in transgenic salvia miltiorrhiza. Plant Cell Physiol 57, 1593-1609. doi:10.1093/pcp/pcw084 |
| 50 | *AT5G51990* | 835274 | Improve drought resistance | Haake, V., Cook, D., Riechmann, J.L., Pineda, O., and Thomashow, M.F., et al. (2002). Transcription factor CBF4 is a regulator of drought adaptation in Arabidopsis. Plant Physiol 130, 639-648. doi:10.1104/pp.006478 |
| 51 | *AT1G01250* | 839322 |  |  |
| 52 | *AT1G46768* | 841117 | Regulate abiotic stress | Dong, C.J., and Liu, J.Y. (2010). The Arabidopsis EAR-motif-containing protein RAP2.1 functions as an active transcriptional repressor to keep stress responses under tight control. BMC Plant Biol 10, 47. doi:10.1186/1471-2229-10-47 |
| 53 | *AT4G06746* | 826148 |  |  |
| 54 | *AT2G23340* | 816866 |  |  |
| 55 | *[AT3G50260](https://bar.utoronto.ca/thalemine/portal.do?externalids=AT3G50260)* | 824188 | Improve low temperature resistance | Tsutsui, T., Kato, W., Asada, Y., Sako, K., and Sato, T., et al. (2009). DEAR1, a transcriptional repressor of DREB protein that mediates plant defense and freezing stress responses in Arabidopsis. J Plant Res 122, 633-643. doi:10.1007/s10265-009-0252-6 |
| 56 | *[AT5G67190](https://bar.utoronto.ca/thalemine/portal.do?externalids=AT5G67190)* | 836854 |  |  |
| 57 | *AT3G11020* | 820273 | Improve the resistance to dehydration and high salt | Nakashima, K., Shinwari, Z.K., Sakuma, Y., Seki, M., and Miura, S., et al. (2000). Organization and expression of two Arabidopsis DREB2 genes encoding DRE-binding proteins involved in dehydration- and high-salinity-responsive gene expression. Plant Mol Biol 42, 657-665. doi:10.1023/a:1006321900483 |
| 58 | *[AT5G05410](https://bar.utoronto.ca/thalemine/portal.do?externalids=AT5G05410)* | 830424 | Improve heat resistance | Mizoi, J., Kanazawa, N., Kidokoro, S., Takahashi, F., and Qin, F., et al. (2019). Heat-induced inhibition of phosphorylation of the stress-protective transcription factor DREB2A promotes thermotolerance of Arabidopsis thaliana. J Biol Chem 294, 902-917. doi:10.1074/jbc.RA118.002662 |
| 59 | *[AT2G46310](https://bar.utoronto.ca/thalemine/portal.do?externalids=AT2G46310)* | 819239 | Mediates the initial cytokinin response | Rashotte, A.M., Mason, M.G., Hutchison, C.E., Ferreira, F.J., and Schaller, G.E., et al. (2006). A subset of Arabidopsis AP2 transcription factors mediates cytokinin responses in concert with a two-component pathway. Proc Natl Acad Sci U S a 103, 11081-11085. doi:10.1073/pnas.0602038103 |
| 60 | *AT3G61630* | 825336 | Negatively regulates leaf senescence | Zwack, P.J., Robinson, B.R., Risley, M.G., and Rashotte, A.M. (2013). Cytokinin response factor 6 negatively regulates leaf senescence and is induced in response to cytokinin and numerous abiotic stresses. Plant Cell Physiol 54, 971-981. doi:10.1093/pcp/pct049 |
| 61 | *[AT4G11140](https://bar.utoronto.ca/thalemine/portal.do?externalids=AT4G11140)* | 826715 | Response to the cytokinin signaling pathway | Cutcliffe, J.W., Hellmann, E., Heyl, A., and Rashotte, A.M. (2011). CRFs form protein-protein interactions with each other and with members of the cytokinin signalling pathway in Arabidopsis via the CRF domain. J Exp Bot 62, 4995-5002. doi:10.1093/jxb/err199 |
| 62 | *[AT1G50640](https://bar.utoronto.ca/thalemine/portal.do?externalids=AT1G50640)* | 841486 |  |  |
| 63 | *[AT3G20310](https://bar.utoronto.ca/thalemine/portal.do?externalids=AT3G20310)* | 821575 | Response to drought stress | Song, C.P., Agarwal, M., Ohta, M., Guo, Y., and Halfter, U., et al. (2005). Role of an Arabidopsis AP2/EREBP-type transcriptional repressor in abscisic acid and drought stress responses. Plant Cell 17, 2384-2396. doi:10.1105/tpc.105.033043 |
| 64 | *[AT1G28370](https://bar.utoronto.ca/thalemine/portal.do?externalids=AT1G28370)* | 839733 | Enhancing plants defense responses to hemibiotrophic bacterial pathogens | Zheng, X., Xing, J., Zhang, K., Pang, X., and Zhao, Y., et al. (2019). Ethylene response factor ERF11 activates BT4 transcription to regulate immunity to pseudomonas syringae. Plant Physiol 180, 1132-1151. doi:10.1104/pp.18.01209 |
| 65 | *AT2G44840* | 819093 | Control lateral root development | Lv, B., Wei, K., Hu, K., Tian, T., and Zhang, F., et al. (2021). MPK14-mediated auxin signaling controls lateral root development via ERF13-regulated very-long-chain fatty acid biosynthesis. Mol Plant 14, 285-297. doi:10.1016/j.molp.2020.11.011 |
| 66 | *[AT5G47220](https://bar.utoronto.ca/thalemine/portal.do?externalids=AT5G47220)* | 834768 | Modulates cellular response in the reoxidation stage after anoxic treatment | Tsai, K.J., Chou, S.J., and Shih, M.C. (2014). Ethylene plays an essential role in the recovery of Arabidopsis during post-anaerobiosis reoxygenation. Plant Cell Environ 37, 2391-2405. doi:10.1111/pce.12292 |
| 67 | *LOC_Os01g21120* | 4327287 |  |  |
| 68 | *LOC_Os06g09390* | 4340383 | Improve drought resistance | Lee, D.K., Jung, H., Jang, G., Jeong, J.S., and Kim, Y.S., et al. (2016). Overexpression of the OsERF71 transcription factor alters rice root structure and drought resistance. Plant Physiol 172, 575-588. doi:10.1104/pp.16.00379 |
| 69 | *LOC_Os03g08470* | 4331845 | Improve drought resistance | Xiong, H., Yu, J., Miao, J., Li, J., and Zhang, H., et al. (2018). Natural variation in OsLG3 increases drought tolerance in rice by inducing ROS scavenging. Plant Physiol 178, 451-467. doi:10.1104/pp.17.01492 |
| 70 | *LOC_Os07g42510* | 4343912 |  |  |
| 71 | *LOC_Os07g47790* | 4344266 |  |  |
| 72 | *LOC_Os04g46250* | 4336573 |  |  |
| 73 | *LOC_Os06g06970* | 4340258 |  |  |
| 74 | *LOC_Os09g35020* | 4347619 |  |  |
| 75 | *LOC_Os02g13710* | 107280052 |  |  |
| 76 | *LOC_Os06g36000* | 107278321 |  |  |
| 77 | *LOC_Os02g43940* | 4330202 |  |  |
| 78 | *LOC_Os02g54050* | 107278604 |  |  |
| 79 | *LOC_Os08g35240* | 107275353 |  |  |
| 80 | *LOC_Os04g55520* | 4337215 |  |  |
| 81 | *LOC_Os02g46510* | 4330379 |  |  |
| 82 | *LOC_Os12g10560* | 4351755 |  |  |
| 83 | *LOC_Os09g25600* | 4347101 |  |  |
| 84 | *LOC_Os10g26590* | 4348988 |  |  |
| 85 | *LOC_Os03g07940* | 9268480 |  |  |
| 86 | *LOC_Os03g37710* | 4333307 |  |  |
| 87 | *LOC_Os03g56050* | 4334257 |  |  |
| 88 | *LOC_Os07g03250* | 4342308 |  |  |
| 89 | *LOC_Os02g10760* | 4328653 |  |  |
| 90 | *LOC_Os02g55380* | 4331013 |  |  |
| 91 | *LOC_Os05g27930* | 4338484 | Improve salt tolerance | Jing, P., Zou, J., Kong, L., Hu, S., and Wang, B., et al. (2016). OsCCD1, a novel small calcium-binding protein with one EF-hand motif, positively regulates osmotic and salt tolerance in rice. Plant Sci 247, 104-114. doi:10.1016/j.plantsci.2016.03.011 |
| 92 | *LOC_Os03g07830* | 112938276 |  |  |
| 93 | *LOC_Os04g44670* | 4336472 |  |  |
| 94 | *LOC_Os02g51670* | 4330752 |  |  |
| 95 | *LOC_Os08g31580* | 4345541 |  |  |
| 96 | *LOC_Os09g20350* | 4346884 |  |  |
| 97 | *LOC_Os01g12440* | 4326729 |  |  |
| 98 | *LOC_Os01g46870* | 4324188 |  |  |
| 99 | *LOC_Os07g47330* | 4344233 | Regulate grain size | Ren, D., Hu, J., Xu, Q., Cui, Y., and Zhang, Y., et al. (2018). FZP determines grain size and sterile lemma fate in rice. J Exp Bot 69, 4853-4866. doi:10.1093/jxb/ery264 |
| 100 | *LOC_Os02g43790* | 4330189 |  |  |
